# Supplementary figures and images for: Structurally related but genetically unrelated antibody lineages converge on an immunodominant HIV-1 Env neutralizing determinant following trimer immunization
Source: PLoS Pathog. 2021 Sep 24;17(9):e1009543. doi: 10.1371/journal.ppat.1009543 (PMC8494329; doi:10.1371/journal.ppat.1009543)

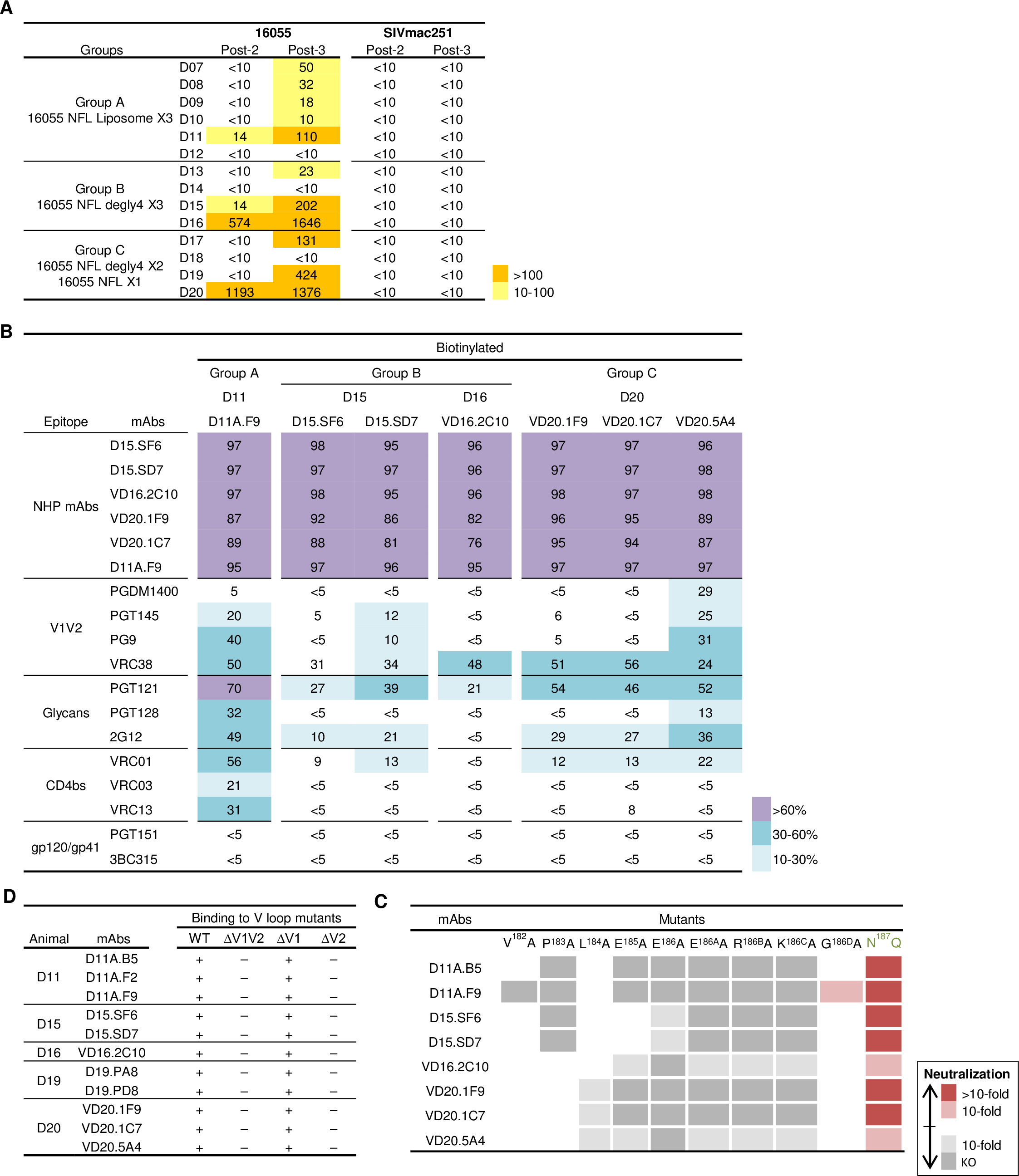

Supplement: S1 Fig — (A) Serum neutralization after 2 (post-2) and 3 (post-3) immunization of each animal. Numbers indicate ID50. (B) Cross-competition binding of biotinylated NHP mAbs to 16055 NFL TD CC trimers (His-captured) in the presence of non-biotinylated mAb competitors (left column) as assessed by ELISA. Percent competition was determined based on the absorbance measured with 200 μg/ml competitor or 10 μg/ml NHP mAbs present and 0% competition being the absorbance measured with no competitor present. (C) Binding of NHP mAbs to 16055 gp120 V loop variants as measured by ELISA: WT, wild-type; ΔV1V2 (Δ126–197); ΔV1 (≥Δ134–153; ΔV2 (Δ159–193); +, binding;–, no binding. (D) Specificity and relative neutralization potencies of NHP mAbs against a panel of V2 point mutant viruses (residues 182–187 were each mutated to Ala, except for N187Q) compared to wild type. Enhanced potencies as measured by IC50 values are highlighted in red (> 10-fold) and pink (10-fold); decreased potencies in grey, knock-out (KO) mutations in dark grey. (TIF) [file ppat.1009543.s001.tif]

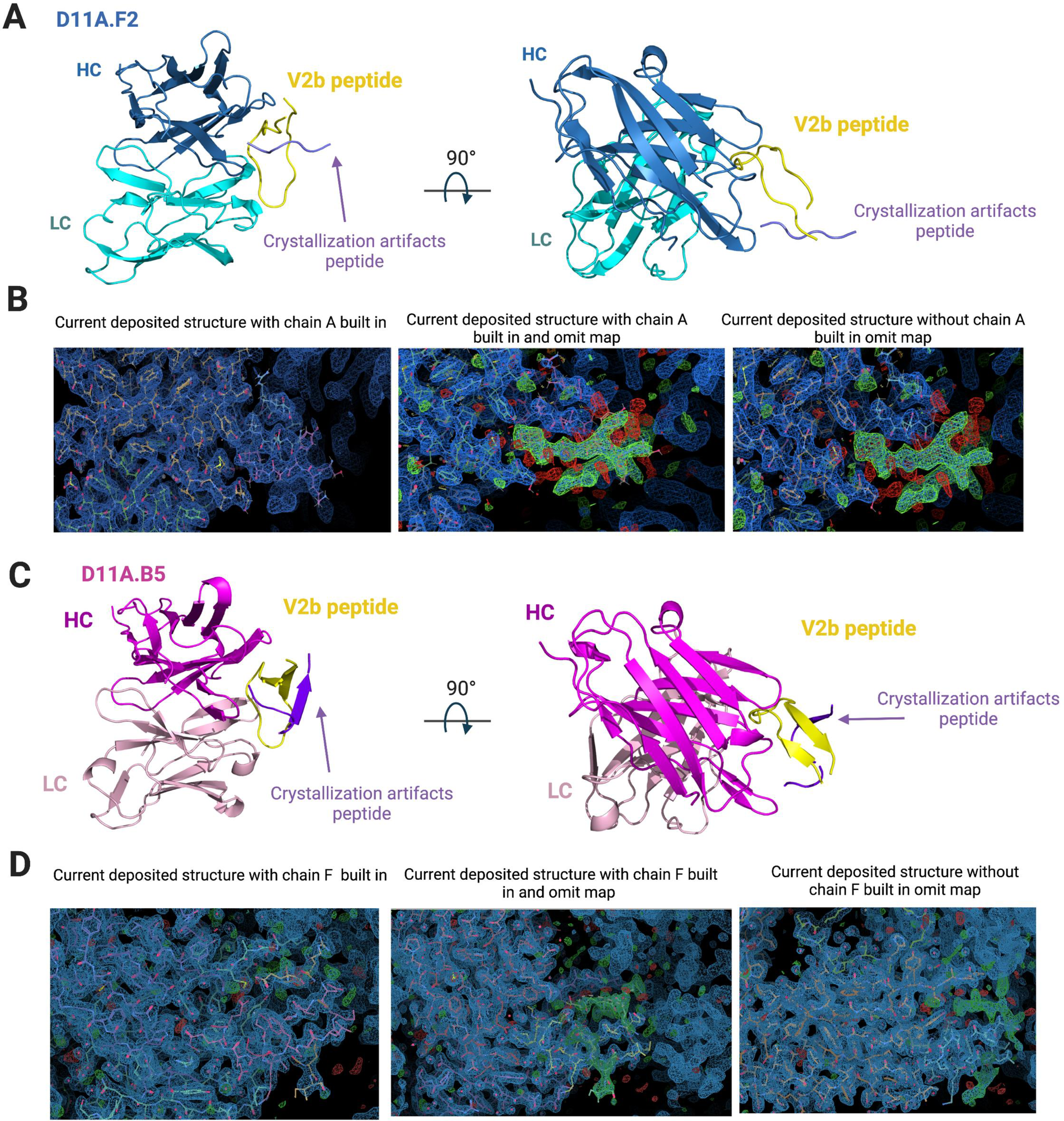

Supplement: S2 Fig — (A) Side and top view of D11A.F2 Fab (Heavy chain, sky blue; Light chain, cyan) bound to the V2b peptide (yellow) and crystallization artifacts peptide (purple). (B) 2Fo-Fc and Fo-Fc electron density showing clear density for the artefact peptide. (C) Side and top view of D11A.B5 Fab (Heavy chain, magenta; Light chain, light pink) bound to the V2b peptide (yellow) and crystallization artifacts peptide (purple). (D) 2Fo-Fc and Fo-Fc electron density showing clear density for the artefact peptide. (TIF) [file ppat.1009543.s002.tif]

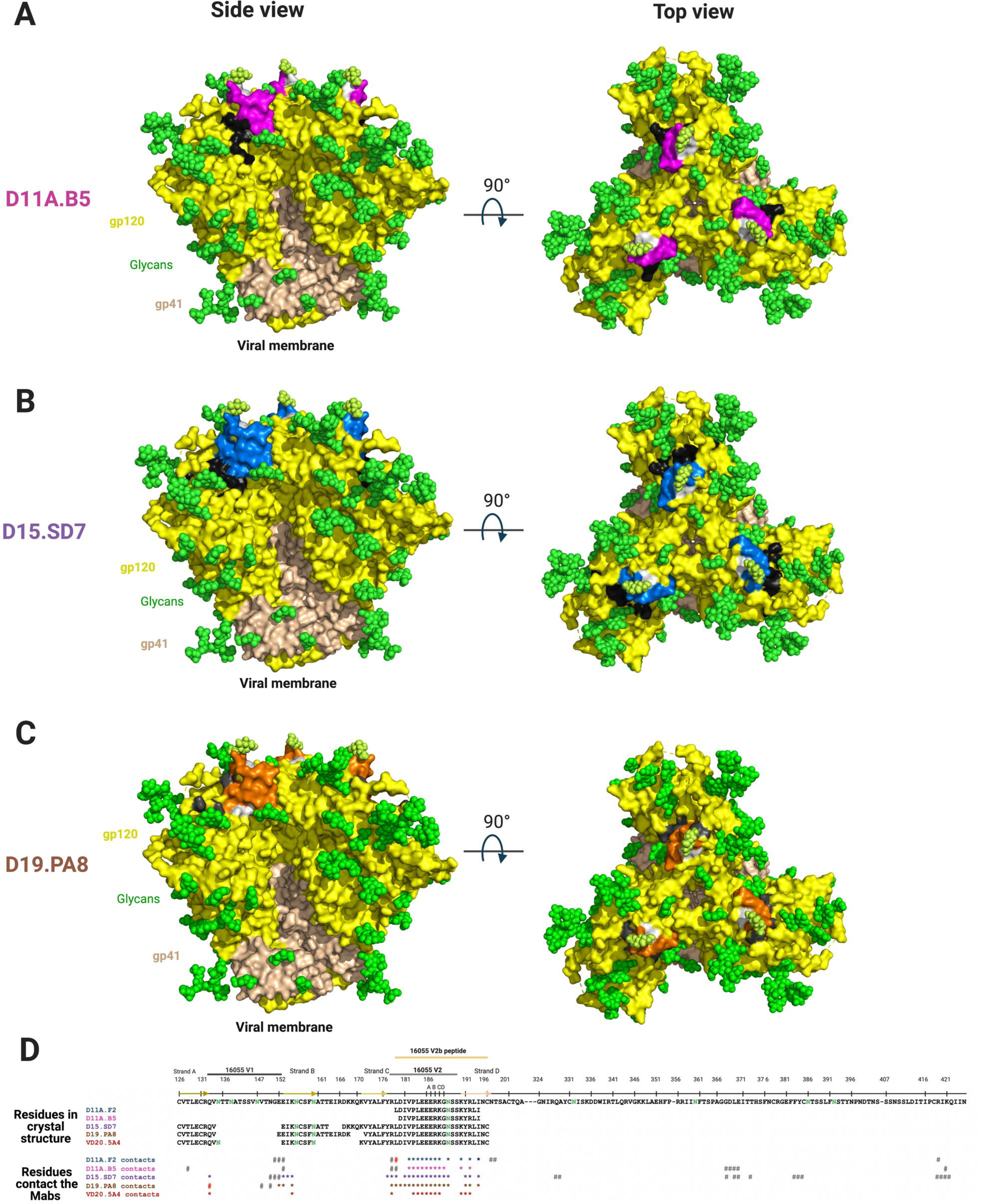

Supplement: S3 Fig — Side and top view of (A) D11A.B5 (magenta), (B) D15.SD7 (blue), and (C) D19.PA8 (orange) epitopes as defined in the crystal structures; residues showing interactions with the trimer that are not ordered/included in the crystal structures are shown in black. (D) Sequence of 16055 gp120 listing residues present/ordered in the crystal structures. Residues within 5Å of the mAbs are shown with asterisks underneath the sequence, residues modeled to interact with the trimer that are absent or disordered in the crystal structures are indicated with a grey # while those present in the crystal structures but only show modeled interactions to the trimer are shown in red #. (TIF) [file ppat.1009543.s003.tif]

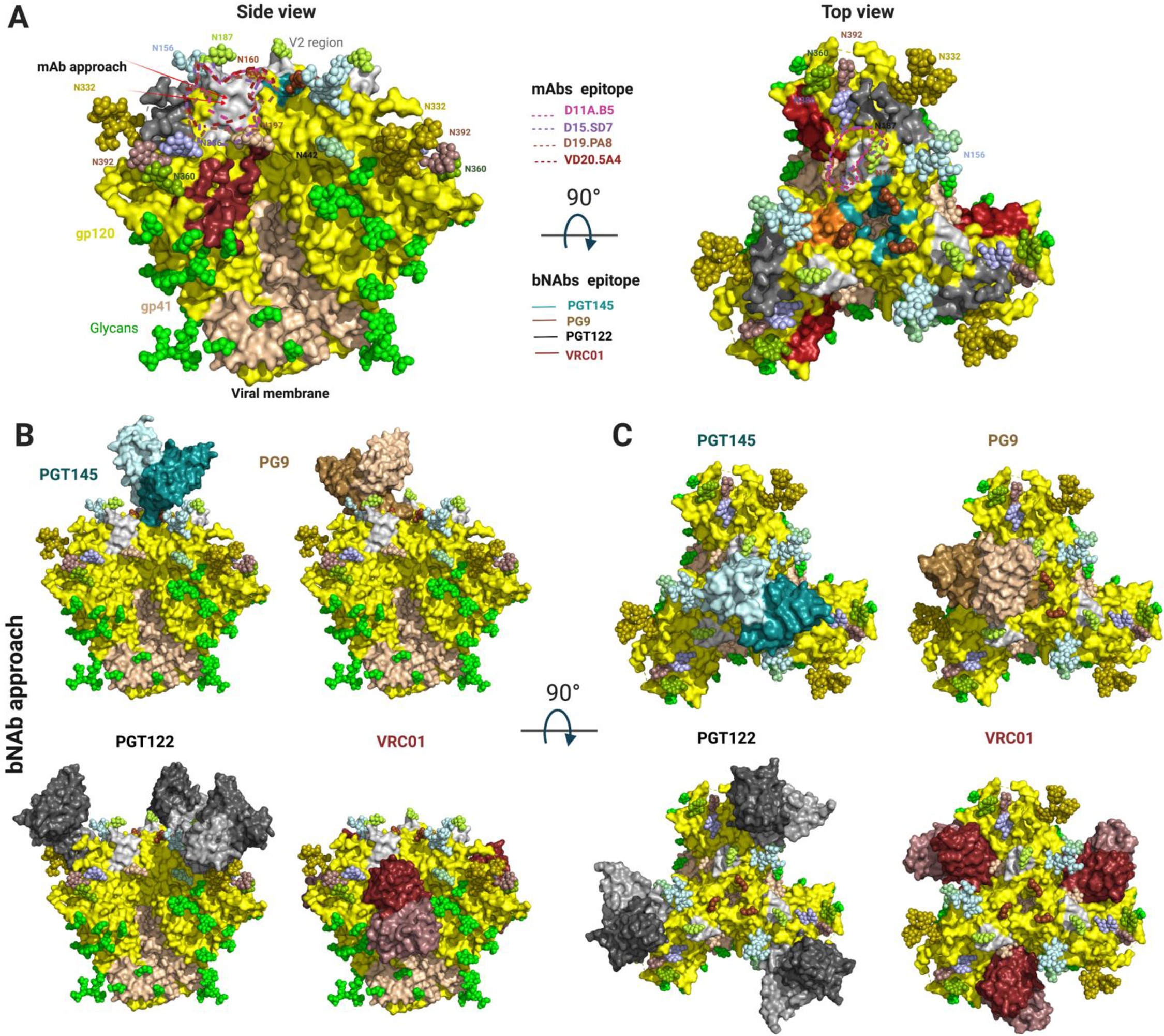

Supplement: S4 Fig — (A) Side and top view surface representation of 16055 NFL (PDB:5UM8) color-coded and labeled as mentioned earlier (Figs 3 and 7). Epitopes targeted by the bNAbs PGT145 (Heavy chain, deepteal; Light chain, light teal)(PDB:5V8L), PG9 (Heavy chain, tv orange; Light chain, wheat)(PDB: 3U2S), PTG122 (Heavy chain, dark gray; Light chain, light gray) and VRC01 (Heavy chain, firebrick; Light chain, light firebrick) (PDB: 5FYK) and our Autologous mAbs (D11A.B5, D15.SD7, D19.PA8, and VD20.5A4) are highlighted and color coded. (B) Side view and (C) Top view superpositions of the bNAbs antibodies structures of PGT145, PG9, PTG122 and VRC01 onto the 16055 NFL trimer, showing how they target their epitopes. Trimer and mAbs are shown in surface representation. (TIF) [file ppat.1009543.s004.tif]
