# Supplementary material for: Structurally related but genetically unrelated antibody lineages converge on an immunodominant HIV-1 Env neutralizing determinant following trimer immunization
Source: PLoS Pathog. 2021 Sep 24;17(9):e1009543. doi: 10.1371/journal.ppat.1009543 (PMC8494329; doi:10.1371/journal.ppat.1009543)
Supplement: S1 Table — (DOCX) [file ppat.1009543.s005.docx]

**S1 Table. Data collection and refinement statistics for crystal structures.**

|  | **D11A.B5 with V2b** | **D11A.F2 with V2b** | **D15.SD7 with 1FD6** | **D19.PA8 with 1FD6** | **VD20.5A4 with 1FD6** |
| --- | --- | --- | --- | --- | --- |
| **Data collection** |  |  |  |  |  |
| Space group | P 2_1_ 2_1_ 2_1_ | P 6_1_ 2 2 | P 2_1_ 2_1_ 2_1_ | P 2_1_ 2_1_ 2_1_ | P4_1_ 2 2 |
| Cell dimensions |  |  |  |  |  |
| *a*, *b*, *c* (Å) | 64.78, 74.78, 101.45 | 130.39, 130.39, 170.9 | 74.41, 89.81, 172.79 | 73.69, 97.89, 168.01 | 106.57, 106.57, 128.93 |
| *α, β, γ* (°) | 90.00, 90.00, 90.00 | 90.00, 90.00, 120.00 | 90.00, 90.00, 90.00 | 90.00, 90.00, 90.00 | 90.00, 90.00, 90.00 |
| Resolution (Å) | 60.2- 2.0 | 112.9-2.8 | 48.5- 2.8 | 84.6-1.9 | 106.6 – 2.9 |
| *R*_merge_^a^ | 0.039 (0.210) | 0.077 (0.511) | 0.927 (0.114) | 0.074 (1.300) | 0.070 (0.646) |
| <I/σ(I)> | 7.64 (2.19) | 11.90 (2.00) | 12.03 (1.05) | 18.42 (1.03) | 10.88 2.30) |
| CC_1/2_ | 0.997 (0.885) | 0.720 (0.744) | 0.998 (0.853) | 0.991 (0.563) | 1.007 (0.980) |
| Completeness | 99.33 (96.5) | 99.66 (98.9) | 99.9 (99.0) | 99.2 (98.8) | 100 (99.9) |
| Redundancy | 6.4 (5.7) | 22.2 (22.2) | 6.2 (4.2) | 7.1 (7.1) | 12.7 (11.6) |
| **Refinement** |  |  |  |  |  |
| Resolution (Å) | 41.98 - 2.0 (2.072 - 2.0) | 68.14 - 2.8 (2.9 – 2.8) | 47.75 - 2.795 (2.895 - 2.795) | 31.9 - 1.904 (1.972 - 1.904) | 48.98 – 2.87 (2.973- 2.87) |
| No. unique reflections | 33786 (3230) | 21734 (2121) | 28184 (1943) | 84546 (2986) | 16556 (977) |
| *R*_work_^b^/*R*_free_^c^ | 18.94/23.37 (27.04/31.42) | 21.45/25.38 (26.57/30.58) | 22.41/28.33 (28.35/35.94) | 18.92/22.04 (27.31/28.61) | 23.91/27.55 (29.96/38.70) |
| No. atoms | 3649 | 3600 | 8204 | 8387 | 3670 |
| Protein | 3359 | 3417 | 8057 | 7377 | 3582 |
| Water | 283 | 96 | 71 | 954 | 48 |
| Ligand | 7 | 87 | 76 | 56 | 40 |
| B-factors (Å^2^) | 40.14 | 58.22 | 56.01 | 29.84 | 55.78 |
| Protein | 39.85 | 57.64 | 55.93 | 29.14 | 55.70 |
| Water | 43.22 | 53.23 | 41.26 | 34.50 | 31.94 |
| Ligand | 57.84 | 86.55 | 78.37 | 42.03 | 91.47 |
| RMS bond length (Å) | 0.005 | 0.008 | 0.005 | 0.004 | 0.016 |
| RMS bond angle (°) | 1.10 | 0.89 | 0.75 | 0.76 | 1.38 |
| **Ramachadran Plot Statistics^d^** | | | | | |
| Residues | 448 | 453 | 1061 | 971 | 478 |
| Most Favored region | 97.00 | 97.29 | 97.57 | 98.00 | 96.12 |
| Allowed Region | 2.76 | 2.71 | 2.43 | 2.40 | 3.88 |
| Clashscore | 4.90 | 0.87 | 0.69 | 1.92 | 5.28 |
| **PDB ID** | 6VJN | 6XLZ | 6WIT | 6WAS | 6XSN |

^a^R_merge_ [∑_h_∑_i_|*I*_h_ – *I*_hi_|/∑_h_∑_i_*I*_hi_] where *I*_h_ is the mean of *I*_hi_ observations of reflection *h*. Numbers in parenthesis represent highest resolution shell. ^b^R_factor_ and ^c^R­_free_ = ∑||F_obs_| - |F_calc_|| / ∑|F_obs_| x 100 for 95% of recorded data (R_factor_) or 5% data (R_free_). ^d^MolProbity reference
