# Supplementary material for: Structurally related but genetically unrelated antibody lineages converge on an immunodominant HIV-1 Env neutralizing determinant following trimer immunization
Source: PLoS Pathog. 2021 Sep 24;17(9):e1009543. doi: 10.1371/journal.ppat.1009543 (PMC8494329; doi:10.1371/journal.ppat.1009543)
Supplement: S2 Table — (DOCX) [file ppat.1009543.s006.docx]

**S2 Table. Detailed interactions of D11A.F2 with 16055 V2b peptide (from PISA web server). (**[**http://www.ebi.ac.uk/msd-srv/prot_int/cgi-bin/piserver**](http://www.ebi.ac.uk/msd-srv/prot_int/cgi-bin/piserver)**)**

| **a. Detailed interactions of D11A.F2 heavy chain (HC) with 16055 V2b peptide** | | | | | | | | | | | | |
| --- | --- | --- | --- | --- | --- | --- | --- | --- | --- | --- | --- | --- |
|  |  |  |  |  |  |  |  |  |  |  |  |  |
| **D11A.F2 HC** | **HSDC** | **ASA** | **BSA** |  | **V2b peptide** | **HSDC** | **ASA** | **BSA** |  | **Hydrogen Bonds** | |  |
| **H:ARG  30** |  | 180.66 | 17.29  \| |  | **P:VAL 182** |  | 50.39 | 31.32  \|\|\|\|\|\|\| |  | **D11A.F2 HC** | **Dist. [Å]** | **V2b peptide** |
| **H:ASP  31** | HS | 83.35 | 71.82  \|\|\|\|\|\|\|\|\| |  | **P:PRO 183** |  | 69.36 | 50.46  \|\|\|\|\|\|\|\| |  | H:SER  53[ H  ] | 2.2 | P:LEU 184[ O  ] |
| **H:TYR  32** |  | 88.67 | 48.17  \|\|\|\|\|\| |  | **P:LEU 184** | H | 110.39 | 81.49  \|\|\|\|\|\|\|\| |  | H:ASN  35[HD21] | 2.18 | P:GLU 186[ OE1] |
| **H:TRP  33** |  | 89.5 | 75.49  \|\|\|\|\|\|\|\|\| |  | **P:GLU 185** |  | 82 | 54.68  \|\|\|\|\|\|\| |  | H:ASP  31[ OD1] | 1.8 | P:ARG 192[ HE ] |
| **H:ASN  35** | H | 19.28 | 6.35  \|\|\|\| |  | **P:GLU 186** | HS | 127.66 | 79.97  \|\|\|\|\|\|\| |  | H:ASP  31[ OD2] | 2.03 | P:ARG 192[HH21] |
| **H:ARG  50** | S | 67.05 | 32.88  \|\|\|\|\| |  | **P:GLU 186A** |  | 113.06 | 31.72  \|\|\| |  |  |  |  |
| **H:ASP  52** |  | 24.95 | 17.37  \|\|\|\|\|\|\| |  | **P:ARG 186B** |  | 144.49 | 8.15  \| |  | **Salt Bridges** | |  |
| **H:SER  53** | H | 50.69 | 24.78  \|\|\|\|\| |  | **P:LYS 190** |  | 139.11 | 24.12  \|\| |  | **D11A.F2 HC** | **Dist. [Å]** | **V2b peptide** |
| **H:VAL  54** |  | 116.85 | 70.06  \|\|\|\|\|\| |  | **P:ARG 192** | HS | 136.16 | 42.13  \|\|\|\| |  | H:ARG  50[ NH2] | 3.96 | P:GLU 186[ OE1] |
| **H:ASN  56** |  | 67.8 | 7.57  \|\| |  | **P:ILE 194** |  | 100.3 | 6.87  \| |  | H:ARG  50[ NE ] | 3.39 | P:GLU 186[ OE1] |
| **H:TYR  58** |  | 92.52 | 9.69  \|\| |  |  |  |  |  |  | H:ARG  50[ NH1] | 3.62 | P:GLU 186[ OE2] |
| **H:SER 100** |  | 123.08 | 7.98  \| |  |  |  |  |  |  | H:ARG  50[ NH2] | 3.27 | P:GLU 186[ OE2] |
| **H:ALA 100A** |  | 75.37 | 1.34  \| |  |  |  |  |  |  | H:ARG  50[ NE ] | 3.53 | P:GLU 186[ OE2] |
| **H:CYS 100B** |  | 58.57 | 14.21  \|\|\| |  |  |  |  |  |  | H:ASP  31[ OD1] | 2.65 | P:ARG 192[ NE ] |
| **H:ASP 101** |  | 64.3 | 3.20  \| |  |  |  |  |  |  | H:ASP  31[ OD2] | 3.53 | P:ARG 192[ NE ] |
|  |  |  |  |  |  |  |  |  |  | H:ASP  31[ OD1] | 3.43 | P:ARG 192[ NH2] |
|  |  |  |  |  |  |  |  |  |  | H:ASP  31[ OD2] | 2.86 | P:ARG 192[ NH2] |
| **b. Detailed interactions of D11A.F2 light chain (LC) with 16055 V2b peptide** | | | | | | | | | |  |  |  |
|  |  |  |  |  |  |  |  |  |  |  |  |  |
| **D11A.F2 LC** | **HSDC** | **ASA** | **BSA** |  | **V2b peptide** | **HSDC** | **ASA** | **BSA** |  | **Hydrogen Bonds** | | |
| **L:ASP  29** |  | 78.61 | 27.71  \|\|\|\| |  | **P:GLU 186** |  | 127.66 | 34.13  \|\|\| |  | **D11A.F2 LC** | **Dist. [Å]** | **V2b peptide** |
| **L:SER  30** |  | 69.44 | 22.15  \|\|\|\| |  | **P:GLU 186A** | HS | 113.06 | 47.48  \|\|\|\|\| |  | L:ARG  50[HH22] | 2.07 | P:GLU 186A[ OE2] |
| **L:GLU  31** | HS | 49.08 | 37.66  \|\|\|\|\|\|\|\| |  | **P:ARG 186B** | HS | 144.49 | 109.39  \|\|\|\|\|\|\|\| |  | L:GLU  31[ OE2] | 2.26 | P:ARG 186B[ HE ] |
| **L:TYR  32** |  | 120.85 | 62.12  \|\|\|\|\|\| |  | **P:LYS 186C** | HS | 200.44 | 149.66  \|\|\|\|\|\|\|\| |  | L:GLU  31[ OE2] | 2.00 | P:ARG 186B[HH21 |
| **L:ARG  50** | HS | 136.95 | 40.60  \|\|\| |  | **P:GLY 186D** |  | 84.64 | 0.15  \| |  | L:ASP  51[ OD2] | 1.89 | P:LYS 186C[ HZ2] |
| **L:ASP  51** | HS | 33.06 | 20.04  \|\|\|\|\|\|\| |  | **P:ASN 187** |  | 105.10 | 21.87  \|\|\| |  |  |  |  |
| **L:ILE  66** |  | 57.76 | 3.83  \| |  |  |  |  |  |  | **Salt Bridges** |  |  |
| **L:ALA  91** |  | 11.54 | 9.04  \|\|\|\|\|\|\|\| |  |  |  |  |  |  | **D11A.F2 LC** | **Dist. [Å]** | **V2b peptide** |
| **L:TYR  95** |  | 168.92 | 59.23  \|\|\|\| |  |  |  |  |  |  | L:ARG  50[ NH2] | 3.92 | P:GLU 186A[ OE1] |
| **L:TRP  96** |  | 156.74 | 26.61  \|\| |  |  |  |  |  |  | L:ARG  50[ NH1] | 3.76 | P:GLU 186A[ OE2] |
|  |  |  |  |  |  |  |  |  |  | L:ARG  50[ NH2] | 2.93 | P:GLU 186A[ OE2] |
|  |  |  |  |  |  |  |  |  |  | L:GLU  31[ OE1] | 3.77 | P:ARG 186B[ NE ] |
|  |  |  |  |  |  |  |  |  |  | L:GLU  31[ OE2] | 3.00 | P:ARG 186B[ NE ] |
|  |  |  |  |  |  |  |  |  |  | L:GLU  31[ OE2] | 2.80 | P:ARG 186B[ NH2] |
|  |  |  |  |  |  |  |  |  |  | L:ASP  51[ OD2] | 2.77 | P:LYS 186C[ NZ ] |
|  |  |  |  |  |  |  |  |  |  |  |  |  |
|  |  |  |  |  |  |  |  |  |  |  |  |  |
|  |  |  |  |  |  |  |  |  |  |  |  |  |
| **c. Detailed interactions of D11A.F2 heavy chain (HC) with artifice 16055 V2b peptide** | | | | | | | | | | | | |
|  |  |  |  |  |  |  |  |  |  |  |  |  |
| **D11A.F2 HC** | **HSDC** | **ASA** | **BSA** |  | **V2b peptide** | **HSDC** | **ASA** | **BSA** |  | **Hydrogen Bonds** |  |  |
| **H:TYR  32** |  | 88.67 | 33.08  \|\|\|\| |  | **A:LEU 193** |  | 177.16 | 37.50  \|\|\| |  | **D11A.F2 LC** | **Dist. [Å]** | **V2b peptide** |
| **H:CYS  97** |  | 40.37 | 13.72  \|\|\|\| |  | **A:ILE 194** |  | 183.24 | 84.08  \|\|\|\|\| |  | L:ASP  60[ OD2] | 3.67 | A:ARG 192[ NH2] |
| **H:GLY  99** |  | 52.14 | 18.74  \|\|\|\| |  |  |  |  |  |  |  |  |  |
| **H:SER 100** |  | 123.08 | 50.03  \|\|\|\|\| |  |  |  |  |  |  |  |  |  |
| **H:CYS 100B** |  | 58.57 | 5.33  \| |  |  |  |  |  |  |  |  |  |
|  |  |  |  |  |  |  |  |  |  |  |  |  |
|  |  |  |  |  |  |  |  |  |  |  |  |  |
| **d. Detailed interactions of D11A.F2 light chain (LC) with artifice 16055 V2b peptide** | | | | | | | | | | | | |
|  |  |  |  |  |  |  |  |  |  |  |  |  |
| **D11A.F2 LC** | **HSDC** | **ASA** | **BSA** |  | **V2b peptide** | **HSDC** | **ASA** | **BSA** |  |  |  |  |
| L:THR  18 |  | 75.83 | 36.85  \|\|\|\|\| |  | A:ARG 192 | S | 212.38 | 73.13  \|\|\|\| |  |  |  |  |
| L:ASP  60 | S | 116.19 | 20.23  \|\| |  | A:ILE 194 |  | 183.24 | 43.93  \|\|\| |  |  |  |  |
| L:ARG  61 |  | 36.75 | 15.38  \|\|\|\|\| |  |  |  |  |  |  |  |  |  |
| L:SER  76 |  | 44.94 | 29.14  \|\|\|\|\|\|\| |  |  |  |  |  |  |  |  |  |
| L:GLY  77 |  | 30.50 | 6.66  \|\|\| |  |  |  |  |  |  |  |  |  |
|  |  |  |  |  |  |  |  |  |  |  |  |  |

ASA Accessible Surface Area, Å² BSA Buried Surface Area, Å² |||| Buried area percentage, one bar per 10%
