# Supplementary material for: Structurally related but genetically unrelated antibody lineages converge on an immunodominant HIV-1 Env neutralizing determinant following trimer immunization
Source: PLoS Pathog. 2021 Sep 24;17(9):e1009543. doi: 10.1371/journal.ppat.1009543 (PMC8494329; doi:10.1371/journal.ppat.1009543)
Supplement: S3 Table — (DOCX) [file ppat.1009543.s007.docx]

**S3 Table. Detailed interactions of D11A.B5 with 16055 V2b peptide (from PISA web server). (http://www.ebi.ac.uk/msd-srv/prot_int/cgi-bin/piserver)**

| **a.     Detailed interactions of D11A.B5 heavy chain (HC) with 16055 V2b peptide** | | | | | | | | | | | | |
| --- | --- | --- | --- | --- | --- | --- | --- | --- | --- | --- | --- | --- |
|  |  |  |  |  |  |  |  |  |  |  |  |  |
| **D11A.B5 HC** | **HSDC** | **ASA** | **BSA** |  | **V2b peptide** | **HSDC** | **ASA** | **BSA** |  | **Hydrogen Bonds** | | |
| **H:SER  30** |  | 54.21 | 0.67  \| |  | **G:VAL 182** |  | 60.35 | 35.74  \|\|\|\|\|\| |  | **D11A.B5 HC** | **Dist. [Å]** | **V2b peptide** |
| **H:ASP  31** | HS | 94.90 | 70.41  \|\|\|\|\|\|\|\| |  | **G:PRO 183** | H | 61.11 | 45.66  \|\|\|\|\|\|\|\| |  | H:ASP  31[ OD1] | 3.05 | G:ARG 192[ NE ] |
| **H:TYR  32** | H | 83.91 | 46.91  \|\|\|\|\|\| |  | **G:LEU 184** | H | 111.42 | 80.47  \|\|\|\|\|\|\|\| |  | H:ASP  31[ OD2] | 2.87 | G:ARG 192[ NH2] |
| **H:TRP  33** |  | 85.05 | 72.83  \|\|\|\|\|\|\|\|\| |  | **G:GLU 185** |  | 81.93 | 56.18  \|\|\|\|\|\|\| |  | H:TYR  32[ N  ] | 3.03 | G:PRO 183[ O  ] |
| **H:ARG  50** | S | 73.36 | 35.48  \|\|\|\|\| |  | **G:GLU 186** | S | 126.61 | 77.68  \|\|\|\|\|\|\| |  | H:SER  53[ N  ] | 3.11 | G:LEU 184[ O  ] |
| **H:ASP  52** |  | 24.82 | 21.80  \|\|\|\|\|\|\|\|\| |  | **G:GLU 186A** |  | 104.66 | 22.07  \|\|\| |  | H:ILE  54[ N  ] | 3.51 | G:LEU 184[ O  ] |
| **H:SER  53** | H | 70.96 | 16.40  \|\|\| |  | **G:ARG 186B** |  | 127.45 | 6.49  \| |  |  |  |  |
| **H:ILE  54** | H | 145.76 | 77.29  \|\|\|\|\|\| |  | **G:LYS 190** |  | 140.34 | 28.67  \|\|\| |  | **Salt Bridges** | | |
| **H:ASN  56** |  | 71.85 | 12.03  \|\| |  | **G:ARG 192** | HS | 130.98 | 21.84  \|\| |  | **D11A.B5 HC** | **Dist. [Å]** | **V2b peptide** |
| **H:TYR  58** |  | 89.48 | 6.26  \| |  |  |  |  |  |  | H:ASP  31[ OD1] | 3.05 | G:ARG 192[ NE ] |
| **H:CYS 102** |  | 68.98 | 16.30  \|\|\| |  |  |  |  |  |  | H:ASP  31[ OD2] | 3.62 | G:ARG 192[ NE ] |
| **H:GLU 104** |  | 76.05 | 4.79  \| |  |  |  |  |  |  | H:ASP  31[ OD1] | 3.77 | G:ARG 192[ NH2] |
|  |  |  |  |  |  |  |  |  |  | H:ASP  31[ OD2] | 2.87 | G:ARG 192[ NH2] |
|  |  |  |  |  |  |  |  |  |  | H:ARG  50[ NH2] | 3.32 | G:GLU 186[ OE2] |
|  |  |  |  |  |  |  |  |  |  | H:ARG  50[ NH1] | 3.65 | G:GLU 186[ OE2] |
|  |  |  |  |  |  |  |  |  |  | H:ARG  50[ NE ] | 3.71 | G:GLU 186[ OE2] |
|  |  |  |  |  |  |  |  |  |  |  |  |  |
| **b.     Detailed interactions of D11A.B5 light chain (LC) with16055 V2b peptide.** | | | | | | | | | | | | |
|  |  |  |  |  |  |  |  |  |  |  |  |  |
| **D11A.B5 LC** | **HSDC** | **ASA** | **BSA** |  | **V2b peptide** | **HSDC** | **ASA** | **BSA** |  | **Hydrogen Bonds** | | |
| **L:ILE  28** |  | 0.92 | 0.12  \|\| |  | **G:GLU 186** | H | 126.61 | 45.39  \|\|\|\| |  | **D11A.B5 LC** | **Dist. [Å]** | **V2b peptide** |
| **L:ASP  29** |  | 75.06 | 28.52  \|\|\|\| |  | **G:GLU 186A** |  | 104.66 | 34.46  \|\|\|\| |  | L:GLU  31[ OE2] | 2.54 | G:ARG 186B[ NH2] |
| **L:SER  30** |  | 65.70 | 14.50  \|\|\| |  | **G:ARG 186B** | HS | 127.45 | 100.18  \|\|\|\|\|\|\|\| |  | L:SER  91[ OG ] | 3.60 | G:ARG 186B[ NH2] |
| **L:GLU  31** | HS | 52.09 | 35.04  \|\|\|\|\|\|\| |  | **G:LYS 186C** | HS | 202.15 | 152.41  \|\|\|\|\|\|\|\| |  | L:ASP  51[ OD2] | 2.62 | G:LYS 186C[ NZ ] |
| **L:TYR  32** |  | 102.39 | 56.92  \|\|\|\|\|\| |  | **G:GLY 186D** |  | 84.53 | 1.60  \| |  | L:TYR  95[ OH ] | 3.48 | G:ASN 187[ ND2] |
| **L:ARG  50** |  | 121.61 | 32.56  \|\|\| |  | **G:ASN 187** | H | 106.25 | 15.53  \|\| |  | L:TRP  96[ NE1] | 2.87 | G:GLU 186[ OE1] |
| **L:ASP  51** | HS | 35.02 | 19.73  \|\|\|\|\|\| |  |  |  |  |  |  |  |  |  |
| **L:ILE  66** |  | 51.32 | 3.34  \| |  |  |  |  |  |  | **Salt Bridges** | | |
| **L:SER  91** | H | 14.03 | 9.48  \|\|\|\|\|\|\| |  |  |  |  |  |  | **D11A.B5 LC** | **Dist. [Å]** | **V2b peptide** |
| **L:TYR  95** | H | 169.87 | 63.99  \|\|\|\| |  |  |  |  |  |  | L:GLU  31[ OE1] | 3.62 | G:ARG 186B[ NE ] |
| **L:TRP  96** | H | 159.10 | 28.03  \|\| |  |  |  |  |  |  | L:GLU  31[ OE2] | 3.15 | G:ARG 186B[ NE ] |
|  |  |  |  |  |  |  |  |  |  | L:GLU  31[ OE2] | 2.54 | G:ARG 186B[ NH2] |
|  |  |  |  |  |  |  |  |  |  | L:ASP  51[ OD1] | 3.89 | G:LYS 186C[ NZ ] |
|  |  |  |  |  |  |  |  |  |  | L:ASP  51[ OD2] | 2.62 | G:LYS 186C[ NZ ] |
|  |  |  |  |  |  |  |  |  |  |  |  |  |
|  |  |  |  |  |  |  |  |  |  |  |  |  |
|  |  |  |  |  |  |  |  |  |  |  |  |  |
| **C. Detailed interactions of D11A.B5 heavy chain (HC) with artifice 16055 V2b peptide** | | | | | | | | | | | | |
|  |  |  |  |  |  |  |  |  |  |  |  |  |
| **D11A.B5 HC** | **HSDC** | **ASA** | **BSA** |  | **V2b peptide** | **HSDC** | **ASA** | **BSA** |  | **Hydrogen Bonds** | | |
| **H:TYR  32** | H | 83.91 | 23.76  \|\|\| |  | **F:GLU 186** | H | 174.56 | 80.59  \|\|\|\|\| |  | **D11A.B5 HC** | **Dist. [Å]** | **V2b peptide** |
| **H:ILE  54** |  | 145.76 | 23.10  \|\| |  | **F:GLU 186A** |  | 163.17 | 55.41  \|\|\|\| |  | H:TYR  32[ OH ] | 2.87 | F:GLU 186[ N  ] |
| **H:CYS  97** |  | 35.80 | 2.66  \| |  | **F:ARG 186B** |  | 234.28 | 43.97  \|\| |  | H:GLY 100[ N  ] | 2.77 | F:GLU 186[ O  ] |
| **H:ALA  98** |  | 53.13 | 7.04  \|\| |  | **F:ILE 194** |  | 222.60 | 21.77  \| |  |  |  |  |
| **H:ILE  99** |  | 181.90 | 57.44  \|\|\|\| |  |  |  |  |  |  |  |  |  |
| **H:GLY 100** | H | 85.09 | 35.59  \|\|\|\|\| |  |  |  |  |  |  |  |  |  |
| **H:ARG 101** |  | 54.93 | 5.15  \| |  |  |  |  |  |  |  |  |  |
| **H:CYS 102** |  | 68.98 | 23.83  \|\|\|\| |  |  |  |  |  |  |  |  |  |
|  |  |  |  |  |  |  |  |  |  |  |  |  |
|  |  |  |  |  |  |  |  |  |  |  |  |  |
| **d. Detailed interactions of D11A.B5 light chain (LC) with artifice 16055 V2b peptide** | | | | | | | | | | | | |
|  |  |  |  |  |  |  |  |  |  |  |  |  |
| **D11A.B5 LC** | **HSDC** | **ASA** | **BSA** |  | **V2b peptide** | **HSDC** | **ASA** | **BSA** |  |  |  |  |
| **L:TYR  32** |  | 102.39 | 5.89  \| |  | **F:SER 189** | H | 150.45 | 25.43  \|\| |  |  |  |  |
| **L:ARG  50** | H | 121.61 | 18.49  \|\| |  |  |  |  |  |  |  |  |  |
|  |  |  |  |  |  |  |  |  |  |  |  |  |

ASA Accessible Surface Area, Å² BSA Buried Surface Area, Å² |||| Buried area percentage, one bar per 10%
