# Supplementary material for: Structurally related but genetically unrelated antibody lineages converge on an immunodominant HIV-1 Env neutralizing determinant following trimer immunization
Source: PLoS Pathog. 2021 Sep 24;17(9):e1009543. doi: 10.1371/journal.ppat.1009543 (PMC8494329; doi:10.1371/journal.ppat.1009543)
Supplement: S4 Table — (DOCX) [file ppat.1009543.s008.docx]

**S4 Table. Detailed interactions of D15.SD7 with 16055 V1V2-1FD6 (from PISA web server). (http://www.ebi.ac.uk/msd-srv/prot_int/cgi-bin/piserver)**

| **a. Detailed interactions of D15.SD7 heavy chain (HC) with V1V2-1FD6** | | | | | | | | | | | | | | | | |  |  |
| --- | --- | --- | --- | --- | --- | --- | --- | --- | --- | --- | --- | --- | --- | --- | --- | --- | --- | --- |
|  |  |  |  |  |  | |  |  |  |  |  | |  | |  | | |  |
| **D15.SD7 HC** | **HSDC** | **ASA** | **BSA** |  | **V1V2-1FD6** | | **HSDC** | **ASA** | **BSA** |  | **Hydrogen Bonds** | |  | |  | | |  |
| A:THR  28 |  | 78.61 | 0.50  \| |  | I:LYS 122 | |  | 91.79 | 4.94  \| |  | **D15.SD7 HC** | | **Dist. [Å]** | | **1FD6** | | |  |
| H:THR  30 |  | 56.91 | 2.76  \| |  | I:GLU 153 | |  | 166.40 | 65.34  \|\|\|\| |  | H:ARG  97[ HE ] | | 2.09 | | I:PRO 183[ O  ] | | |  |
| H:ASP  31 |  | 56.26 | 5.52  \| |  | I:TYR 177 | |  | 134.64 | 15.22  \|\| |  | H:VAL 100G[ O  ] | | 2.36 | | I:ARG 178[HH12] | | |  |
| H:GLU  53 |  | 95.85 | 4.17  \| |  | I:ARG 178 | | H | 53.13 | 49.41  \|\|\|\|\|\|\|\|\|\| |  | H:VAL 100G[ O  ] | | 1.92 | | I:ARG 178[HH22] | | |  |
| H:ASP  95 |  | 15.72 | 4.94  \|\|\|\| |  | I:LEU 179 | |  | 150.96 | 100.74  \|\|\|\|\|\|\| |  |  | |  | |  | | |  |
| H:ARG  97 | H | 109.14 | 59.77  \|\|\|\|\|\| |  | I:ILE 181 | |  | 4.67 | 3.69  \|\|\|\|\|\|\|\| |  |  | |  | |  | | |  |
| H:THR 100B |  | 82.48 | 0.12  \| |  | I:VAL 182 | |  | 50.11 | 36.72  \|\|\|\|\|\|\|\| |  |  | |  | |  | | |  |
| H:TYR 100C |  | 203.06 | 44.80  \|\|\| |  | I:PRO 183 | | H | 57.76 | 57.09  \|\|\|\|\|\|\|\|\|\| |  |  | |  | |  | | |  |
| H:PHE 100D |  | 192.25 | 76.66  \|\|\|\| |  | I:LEU 184 | |  | 91.06 | 33.26  \|\|\|\| |  |  | |  | |  | | |  |
| H:SER 100E |  | 45.90 | 34.15  \|\|\|\|\|\|\|\| |  | I:GLU 185 | |  | 87.81 | 20.02  \|\|\| |  |  | |  | |  | | |  |
| H:GLY 100F |  | 35.44 | 29.87  \|\|\|\|\|\|\|\|\| |  | I:GLU 186 | |  | 130.24 | 53.07  \|\|\|\|\| |  |  | |  | |  | | |  |
| H:VAL 100G | H | 98.68 | 55.47  \|\|\|\|\|\| |  | I:GLU 186A | |  | 83.02 | 16.40  \|\| |  |  | |  | |  | | |  |
| H:GLN 100H |  | 69.58 | 20.04  \|\|\| |  | I:ARG 192 | |  | 61.88 | 5.09  \| |  |  | |  | |  | | |  |
| H:TRP 100I |  | 122.54 | 94.64  \|\|\|\|\|\|\|\| |  | I:LYS 233 | |  | 108.32 | 4.20  \| |  |  | |  | |  | | |  |
| H:PRO 100K |  | 88.94 | 22.02  \|\|\| |  |  | |  |  |  |  |  | |  | |  | | |  |
|  |  |  |  |  |  | |  |  |  |  |  | |  | |  | | |  |
|  |  |  |  |  |  | |  |  |  |  |  | |  | |  | | |  |
| **b. Detailed interactions of D15.SD7 light chain (LC) with V1V2-1FD6.** | | | | | | | | | | | | | | | | |  |  |
|  |  |  |  |  |  |  | |  |  |  |  | |  | |  | | |  |
| **D15.SD7 LC** | **HSDC** | **ASA** | **BSA** |  | **V1V2-1FD6** | **HSDC** | | **ASA** | **BSA** |  | **Hydrogen Bonds** | |  | |  | | |  |
| L:ILE  28 | H | 1.44 | 1.10  \|\|\|\|\|\|\|\| |  | I:GLU 153 |  | | 166.40 | 6.12  \| |  | **D15.SD7 LC** | | **Dist. [Å]** | | **V1V2-1FD6** | | |  |
| L:ASP  29 |  | 89.69 | 27.78  \|\|\|\| |  | I:LYS 155 |  | | 37.75 | 5.00  \|\| |  | L:GLU  31[ OE1] | | 2.49 | | I:ARG 186B[ HE ] | | |  |
| L:SER  30 |  | 68.68 | 22.18  \|\|\|\| |  | I:ARG 178 |  | | 53.13 | 3.25  \| |  | L:GLU  31[ OE2] | | 1.87 | | I:ARG 186B[HH21] | | |  |
| L:GLU  31 | HS | 53.39 | 39.71  \|\|\|\|\|\|\|\| |  | I:GLU 186 | HS | | 130.24 | 71.85  \|\|\|\|\|\| |  | L:GLY  93[ O  ] | | 1.89 | | I:ARG 186B[HH22] | | |  |
| L:TYR  32 |  | 112.22 | 78.98  \|\|\|\|\|\|\|\| |  | I:GLU 186A | HS | | 83.02 | 59.66  \|\|\|\|\|\|\|\| |  | L:ASP  51[ OD2] | | 2.36 | | I:LYS 186C[ HZ1] | | |  |
| L:GLN  34 |  | 24.92 | 5.78  \|\|\| |  | I:ARG 186B | HS | | 136.64 | 92.62  \|\|\|\|\|\|\| |  | L:ILE  28[ O  ] | | 2.32 | | I:LYS 186C[ HZ2] | | |  |
| L:LYS  50 | HS | 99.35 | 39.65  \|\|\|\| |  | I:LYS 186C | HS | | 196.82 | 161.83  \|\|\|\|\|\|\|\|\| |  | L:ARG  96[HH21] | | 2.14 | | I:GLU 186[ OE1] | | |  |
| L:ASP  51 | HS | 33.45 | 14.91  \|\|\|\|\| |  | I:GLY 186D |  | | 41.63 | 11.33  \|\|\| |  | L:LYS  50[ HZ1] | | 2.05 | | I:GLU 186A[ OE2] | | |  |
| L:ILE  66 |  | 50.57 | 2.17  \| |  | I:ASN 187 |  | | 88.90 | 10.31  \|\| |  |  | |  | |  | | |  |
| L:GLN  89 |  | 8.08 | 1.30  \|\| |  |  |  | |  |  |  |  | |  | |  | | |  |
| L:PRO  91 |  | 39.36 | 35.47  \|\|\|\|\|\|\|\|\|\| |  |  |  | |  |  |  | **Salt Bridges** | |  | |  | | |  |
| L:GLY  93 | H | 57.29 | 11.16  \|\| |  |  |  | |  |  |  | **D15.SD7 LC** | | **Dist. [Å]** | | **V1V2-1FD6** | | |  |
| L:ARG  94 |  | 221.07 | 13.99  \| |  |  |  | |  |  |  | L:GLU  31[ OE1] | | 3.28 | | I:ARG 186B[ NE ] | | |  |
| L:TYR  95 |  | 192.88 | 0.34  \| |  |  |  | |  |  |  | L:GLU  31[ OE2] | | 3.88 | | I:ARG 186B[ NE ] | | |  |
| L:ARG  96 | HS | 189.93 | 37.54  \|\| |  |  |  | |  |  |  | L:GLU  31[ OE1] | | 3.43 | | I:ARG 186B[ NH2] | | |  |
|  |  |  |  |  |  |  | |  |  |  | L:GLU  31[ OE2] | | 2.69 | | I:ARG 186B[ NH2] | | |  |
|  |  |  |  |  |  |  | |  |  |  | L:ASP  51[ OD1] | | 3.27 | | I:LYS 186C[ NZ ] | | |  |
| **c. Detailed interactions of D15.SD7 light chain (LC) with NAG.** | | | | | | | | | | |  | L:ASP  51[ OD2] | | 2.78 | | I:LYS 186C[ NZ ] | | |
| **D15.SD7 LC** | **HSDC** | **ASA** | **BSA** |  | **NAG** | **HSDC** | | **ASA** | **BSA** | |  | L:ARG  96[ NE ] | | 3.49 | | I:GLU 186[ OE1] | | |
| L:ASP  29 |  | 89.69 | 2.22  \| |  | I:NAG1187 |  | | 351.45 | 98.21  \|\|\| | |  | L:ARG  96[ NH2] | | 2.93 | | I:GLU 186[ OE1] | | |
| L:SER  30 |  | 68.68 | 41.66  \|\|\|\|\|\|\| |  |  |  | |  |  | |  | L:ARG  96[ NH2] | | 3.58 | | I:GLU 186[ OE2] | | |
| L:GLU  31 |  | 53.39 | 13.68  \|\|\| |  |  |  | |  |  | |  | L:LYS  50[ NZ ] | | 2.88 | | I:GLU 186A[ OE2] | | |
| L:GLY  93 |  | 57.29 | 23.16  \|\|\|\|\| |  |  |  | |  |  | |  |  | |  | |  | | |
| L:ARG  94 |  | 221.07 | 11.53  \| |  |  |  | |  |  | |  |  | |  | |  | | |

ASA Accessible Surface Area, Å² BSA Buried Surface Area, Å² |||| Buried area percentage, one bar per 10%
