# Supplementary material for: Structurally related but genetically unrelated antibody lineages converge on an immunodominant HIV-1 Env neutralizing determinant following trimer immunization
Source: PLoS Pathog. 2021 Sep 24;17(9):e1009543. doi: 10.1371/journal.ppat.1009543 (PMC8494329; doi:10.1371/journal.ppat.1009543)
Supplement: S5 Table — (DOCX) [file ppat.1009543.s009.docx]

**S5 Table. Detailed interactions of D19.PA8 with 16055 V1V2-1FD6 (from PISA web server). (**[**http://www.ebi.ac.uk/msd-srv/prot_int/cgi-bin/piserver**](http://www.ebi.ac.uk/msd-srv/prot_int/cgi-bin/piserver)**)**

| **a. Detailed interactions of D19.PA8 heavy chain (HC) with V1V2-1FD6** | | | | | | | | | | | | |
| --- | --- | --- | --- | --- | --- | --- | --- | --- | --- | --- | --- | --- |
|  |  |  |  |  |  |  |  |  |  |  |  |  |
| **D19.PA8 HC** | **HSDC** | **ASA** | **BSA** |  | **V1V2-1FD6** | **HSDC** | **ASA** | **BSA** |  | **Hydrogen Bonds** | | |
| H:GLU  31 |  | 72.48 | 3.88  \| |  | J:ARG 178 |  | 59.28 | 10.14  \|\| |  | **D19.PA8 HC** | **Dist. [Å]** | **V1V2-1FD6** |
| H:TYR  33 |  | 39.46 | 27.90  \|\|\|\|\|\|\|\| |  | J:VAL 182 |  | 44.08 | 15.62  \|\|\|\| |  | H:GLY 100[ O  ] | 2.22 | J:GLU 186A[ H  ] |
| H:ARG  50 | S | 64.90 | 16.12  \|\|\| |  | J:PRO 183 |  | 48.25 | 43.23  \|\|\|\|\|\|\|\|\| |  | H:TYR  52A[ HH ] | 2.11 | J:LEU 184[ O  ] |
| H:TYR  52A | H | 83.32 | 39.99  \|\|\|\|\| |  | J:LEU 184 | H | 92.84 | 41.57  \|\|\|\|\| |  |  |  |  |
| H:VAL  97 |  | 70.60 | 32.18  \|\|\|\|\| |  | J:GLU 185 |  | 88.44 | 27.21  \|\|\|\| |  |  |  |  |
| H:TRP  98 |  | 178.96 | 2.81  \| |  | J:GLU 186 | S | 139.18 | 69.68  \|\|\|\|\|\| |  | **Salt Bridges** |  |  |
| H:THR  99 |  | 23.35 | 1.67  \| |  | J:GLU 186A | H | 70.36 | 30.65  \|\|\|\|\| |  | **D19.PA8 HC** | **Dist. [Å]** | **V1V2-1FD6** |
| H:GLY 100 | H | 48.63 | 41.91  \|\|\|\|\|\|\|\|\| |  | J:ARG 192 |  | 76.40 | 3.70  \| |  | H:ARG  50[ NH1] | 3.99 | J:GLU 186[ OE2] |
| H:TYR 100A |  | 69.13 | 59.69  \|\|\|\|\|\|\|\|\| |  |  |  |  |  |  |  |  |  |
| H:THR 100B |  | 108.78 | 34.33  \|\|\|\| |  |  |  |  |  |  |  |  |  |
|  |  |  |  |  |  |  |  |  |  |  |  |  |
|  |  |  |  |  |  |  |  |  |  |  |  |  |
|  |  |  |  |  |  |  |  |  |  |  |  |  |
| **b. Detailed interactions of D19.PA8 light chain with V1V2-1FD6**. | | | | | | | | | | | | |
|  |  |  |  |  |  |  |  |  |  |  |  |  |
| **D19.PA8 LC** | **HSDC** | **ASA** | **BSA** |  | **V1V2-1FD6** | **HSDC** | **ASA** | **BSA** |  | **Hydrogen Bonds** | | |
| L:ILE  28 |  | 1.65 | 0.49  \|\|\| |  | J:LYS 155 |  | 37.88 | 7.82  \|\|\| |  | **D19.PA8 LC** | **Dist. [Å]** | **V1V2-1FD6** |
| L:ASP  29 | S | 86.81 | 35.65  \|\|\|\|\| |  | J:ARG 178 |  | 59.28 | 4.37  \| |  | L:GLU  31[ OE2] | 2.21 | J:ARG 186B[ HE ] |
| L:ASN  30 |  | 82.98 | 17.83  \|\|\| |  | J:GLU 186 | HS | 139.18 | 69.51  \|\|\|\|\| |  | L:GLU  31[ OE1] | 2.02 | J:ARG 186B[HH21] |
| L:GLU  31 | HS | 66.37 | 49.95  \|\|\|\|\|\|\|\| |  | J:GLU 186A | HS | 70.36 | 39.71  \|\|\|\|\|\| |  | L:ASP  51[ OD2] | 1.95 | J:LYS 186C[ HZ2] |
| L:TYR  32 |  | 114.45 | 71.22  \|\|\|\|\|\|\| |  | J:ARG 186B | HS | 138.27 | 80.84  \|\|\|\|\|\| |  | L:ARG  34[HH12] | 2.29 | J:GLU 186[ OE1] |
| L:ARG  34 | HS | 37.79 | 13.90  \|\|\|\| |  | J:LYS 186C | HS | 203.30 | 162.64  \|\|\|\|\|\|\|\| |  | L:LYS  50[ HZ1] | 1.75 | J:GLU 186A[ OE2] |
| L:LYS  50 | HS | 104.27 | 38.71  \|\|\|\| |  | J:GLY 186D |  | 63.44 | 0.67  \| |  |  |  |  |
| L:ASP  51 | HS | 35.22 | 20.58  \|\|\|\|\|\| |  | J:ASN 187 |  | 77.80 | 7.69  \| |  |  |  |  |
| L:ILE  66 |  | 50.76 | 3.16  \| |  |  |  |  |  |  |  |  |  |
| L:SER  91 |  | 1.89 | 1.52  \|\|\|\|\|\|\|\|\| |  |  |  |  |  |  | **Salt Bridges** |  |  |
| L:PHE  95 |  | 151.21 | 66.17  \|\|\|\|\| |  |  |  |  |  |  | **D19.PA8 LC** | **Dist. [Å]** | **V1V2-1FD6** |
| L:TRP  96 |  | 154.36 | 16.49  \|\| |  |  |  |  |  |  | L:GLU  31[ OE1] | 3.72 | J:ARG 186B[ NE ] |
|  |  |  |  |  |  |  |  |  |  | L:GLU  31[ OE2] | 3.07 | J:ARG 186B[ NE ] |
|  |  |  |  |  |  |  |  |  |  | L:GLU  31[ OE1] | 2.87 | J:ARG 186B[ NH2] |
|  |  |  |  |  |  |  |  |  |  | L:GLU  31[ OE2] | 3.72 | J:ARG 186B[ NH2] |
|  |  |  |  |  |  |  |  |  |  | L:ASP  29[ OD1] | 3.13 | J:LYS 186C[ NZ ] |
|  |  |  |  |  |  |  |  |  |  | L:ASP  51[ OD1] | 3.93 | J:LYS 186C[ NZ ] |
|  |  |  |  |  |  |  |  |  |  | L:ASP  51[ OD2] | 2.79 | J:LYS 186C[ NZ ] |
|  |  |  |  |  |  |  |  |  |  | L:ARG  34[ NH1] | 2.96 | J:GLU 186[ OE1] |
|  |  |  |  |  |  |  |  |  |  | L:LYS  50[ NZ ] | 2.63 | J:GLU 186A[ OE2] |
|  |  |  |  |  |  |  |  |  |  |  |  |  |
| **c. Detailed interactions of D19.PA8 light chain with NAG**. | | | | | | | | | | | | |
|  |  |  |  |  |  |  |  |  |  |  |  |  |
| **D19.PA8 LC** | **HSDC** | **ASA** | **BSA** |  | **NAG** | **HSDC** | **ASA** | **BSA** |  | **Hydrogen Bonds** | | |
| L:ASP  29 |  | 86.81 | 8.47  \| |  | J:NAG1187 | H | 353.10 | 88.28  \|\|\| |  | **D19.PA8 LC** | **Dist. [Å]** | **V1V2-1FD6** |
| L:ASN  30 | H | 82.98 | 46.80  \|\|\|\|\|\| |  |  |  |  |  |  | L:ASN  30[ O  ] | 2.96 | J:NAG1187[ N2 ] |
| L:GLU  31 |  | 66.37 | 15.97  \|\|\| |  |  |  |  |  |  |  |  |  |
| L:ASP  93 |  | 130.68 | 4.68  \| |  |  |  |  |  |  |  |  |  |
|  |  |  |  |  |  |  |  |  |  |  |  |  |
|  |  |  |  |  |  |  |  |  |  |  |  |  |
| **d. Detailed interactions of D19.PA8 heavy chain (HC) with V1V2-1FD6** | | | | | | | |  |  |  |  |  |
|  |  |  |  |  |  |  |  |  |  |  |  |  |
| **D19.PA8 HC** | **HSDC** | **ASA** | **BSA** |  | **V1V2-1FD6** | **HSDC** | **ASA** | **BSA** |  | **Hydrogen Bonds** | | |
| A:GLU  31 |  | 81.48 | 0.61  \| |  | G:ARG 178 |  | 59.50 | 17.55  \|\|\| |  | **D19.PA8 HC** | **Dist. [Å]** | **V1V2-1FD6** |
| A:TYR  33 |  | 37.80 | 28.06  \|\|\|\|\|\|\|\| |  | G:VAL 182 |  | 39.07 | 18.97  \|\|\|\|\| |  | A:GLY 100[ O  ] | 2.13 | G:GLU 186A[ H  ] |
| A:ARG  50 | S | 66.14 | 16.17  \|\|\| |  | G:PRO 183 |  | 45.80 | 44.11  \|\|\|\|\|\|\|\|\|\| |  | A:TYR  52A[ HH ] | 2.06 | G:LEU 184[ O  ] |
| A:TYR  52A | H | 84.69 | 38.45  \|\|\|\|\| |  | G:LEU 184 | H | 93.55 | 39.67  \|\|\|\|\| |  |  |  |  |
| A:VAL  97 |  | 73.35 | 43.32  \|\|\|\|\|\| |  | G:GLU 185 |  | 92.19 | 28.47  \|\|\|\| |  |  |  |  |
| A:TRP  98 |  | 188.12 | 6.01  \| |  | G:GLU 186 | S | 138.74 | 69.94  \|\|\|\|\|\| |  | **Salt Bridges** |  |  |
| A:THR  99 |  | 21.06 | 2.00  \| |  | G:GLU 186A | H | 66.36 | 30.38  \|\|\|\|\| |  | **D19.PA8 HC** | **Dist. [Å]** | **V1V2-1FD6** |
| A:GLY 100 | H | 48.20 | 41.28  \|\|\|\|\|\|\|\|\| |  | G:ARG 192 |  | 73.31 | 0.58  \| |  | A:ARG  50[ NE ] | 3.98 | G:GLU 186[ OE2] |
| A:TYR 100A |  | 67.51 | 58.66  \|\|\|\|\|\|\|\|\| |  |  |  |  |  |  | A:ARG  50[ NH2] | 3.92 | G:GLU 186[ OE2] |
| A:THR 100B |  | 107.85 | 34.42  \|\|\|\| |  |  |  |  |  |  |  |  |  |
|  |  |  |  |  |  |  |  |  |  |  |  |  |
|  |  |  |  |  |  |  |  |  |  |  |  |  |
|  |  |  |  |  |  |  |  |  |  |  |  |  |
|  |  |  |  |  |  |  |  |  |  |  |  |  |
|  |  |  |  |  |  |  |  |  |  |  |  |  |
|  |  |  |  |  |  |  |  |  |  |  |  |  |
| **e. Detailed interactions of D19.PA8 light chain with V1V2-1FD6**. | | | | | | | | | | | | |
|  |  |  |  |  |  |  |  |  |  |  |  |  |
| **D19.PA8 LC** | **HSDC** | **ASA** | **BSA** |  | **V1V2-1FD6** | **HSDC** | **ASA** | **BSA** |  |  | | |
| B:GLU 183 |  | 105.80 | 12.93  \|\| |  | G:GLU 152 |  | 137.97 | 48.52  \|\|\|\| |  |  |  |  |
| B:LYS 186 |  | 118.87 | 63.24  \|\|\|\|\|\| |  | G:GLU 153 |  | 100.05 | 5.79  \| |  |  |  |  |
| B:SER 187 |  | 82.85 | 5.77  \| |  | G:ARG 178 |  | 59.50 | 18.34  \|\|\|\| |  |  |  |  |
| B:ARG 189 |  | 126.35 | 48.84  \|\|\|\| |  | G:LEU 179 |  | 155.27 | 111.82  \|\|\|\|\|\|\|\| |  |  |  |  |
| B:PRO 208 |  | 34.58 | 18.10  \|\|\|\|\|\| |  | G:ILE 181 |  | 2.46 | 1.60  \|\|\|\|\|\|\| |  |  |  |  |
| B:THR 209 |  | 96.74 | 44.48  \|\|\|\|\| |  | G:ILE 194 |  | 105.05 | 35.48  \|\|\|\| |  |  |  |  |
|  |  |  |  |  |  |  |  |  |  |  |  |  |
| **f. Detailed interactions of D19.PA8 light chain with NAG**. | | | | | | | | | | | | |
|  |  |  |  |  |  |  |  |  |  |  |  |  |
| **D19.PA8 LC** | **HSDC** | **ASA** | **BSA** |  | **NAG** | **HSDC** | **ASA** | **BSA** |  |  |  |  |
| B:ASP  29 |  | 83.75 | 8.15  \| |  | G:NAG1187 | H | 354.92 | 106.24  \|\|\| |  |  |  |  |
| B:ASN  30 | H | 77.95 | 44.63  \|\|\|\|\|\| |  |  |  |  |  |  |  |  |  |
| B:GLU  31 |  | 65.77 | 16.86  \|\|\| |  |  |  |  |  |  |  |  |  |
| B:ASP  93 |  | 129.57 | 20.58  \|\| |  |  |  |  |  |  |  |  |  |
|  |  |  |  |  |  |  |  |  |  |  |  |  |

ASA Accessible Surface Area, Å² BSA Buried Surface Area, Å² |||| Buried area percentage, one bar per 10%
