# Supplementary material for: Structurally related but genetically unrelated antibody lineages converge on an immunodominant HIV-1 Env neutralizing determinant following trimer immunization
Source: PLoS Pathog. 2021 Sep 24;17(9):e1009543. doi: 10.1371/journal.ppat.1009543 (PMC8494329; doi:10.1371/journal.ppat.1009543)
Supplement: S6 Table — (DOCX) [file ppat.1009543.s010.docx]

**S6 Table. Detailed interactions of VD20.5A4 with 16055 V1V2-1FD6 (from PISA web server). (http://www.ebi.ac.uk/msd-srv/prot_int/cgi-bin/piserver)**

ASA Accessible Surface Area, Å² BSA Buried Surface Area, Å² |||| Buried area percentage, one bar per 10%
